# Functional Perspective of Leeks: Active Components, Health Benefits and Action Mechanisms

**DOI:** 10.3390/foods12173225

**Published:** 2023-08-27

**Authors:** Tiantian Xie, Qi Wu, Han Lu, Zuomin Hu, Yi Luo, Zhongxing Chu, Feijun Luo

**Affiliations:** 1 Hunan Key Laboratory of Grain-Oil Deep Process and Quality Control, College of Food Science and Engineering, Central South University of Forestry and Technology, Changsha 410004, China; xtt04160530@163.com (T.X.); 17663231179@163.com (Q.W.); lu18365138735@163.com (H.L.); huzuomin97100214@163.com (Z.H.); czxcs2021@163.com (Z.C.); 2 Hunan Key Laboratory of Forestry Edible Resources Safety and Processing, College of Food Science and Engineering, Central South University of Forestry and Technology, Changsha 410004, China; 3 Department of Gastroenterology, Xiangya Hospital, Central South University, Changsha 410008, China; lyzndx0810@163.com

**Keywords:** leeks, bioactive ingredients, anti-inflammation, anti-cancer, anti-oxidation, lowering fat and blood sugar

## Abstract

Leek (*Allium fistulosum* L.), a common and widely used food ingredient, is a traditional medicine used in Asia to treat a variety of diseases. Leeks contain a variety of bioactive substances, including sulfur compounds, dietary fiber, steroid compounds and flavonoid compounds. Many studies have shown that these active ingredients produce the following effects: promotion of blood circulation, lowering of cholesterol, relief of fatigue, anti-inflammation, anti-bacteria, regulation of cell metabolism, anti-cancer, anti-oxidation, and the lowering of fat and blood sugar levels. In this paper, the main bioactive components and biological functions of leeks were systemically reviewed, and the action mechanisms of bioactive components were discussed. As a common food, the health benefits of leeks are not well known, and there is no systematic summary of leek investigations. In light of this, it is valuable to review the recent progress and provide reference to investigators in the field, which will promote future applications and investigations of leeks.

## 1. Introduction

*Allium*, a genus of about 500 species in the *Liliaceae*, is a perennial herb. It was one of the first cultivated vegetables to be used as food [1]. Common members of the genus include garlic (*Allium sativum* L.), onion (*Allium cepa* L.), chive (*Allium schoenoprasum* L.) and leek (*Allium fistulosum* L.) [2,3]. *Allium* is easy to grow and can be preserved for a long time. It is widely cultivated worldwide as a spice and condiment with great culinary and health values [4]. *Allium* contains secondary metabolites such as volatile oils, polysaccharides, phenols and other sulfur-containing compounds. These active substances not only give *Allium* plants a unique odor, but also provide *Allium* plants with many biological functions [5]. The unique smell of different *Allium* plants is mainly related to different S-alk(en)yl-L-cysteine sulfoxide precursors, which are primarily alliin (S-allyl-L-cysteine sulfoxide) and isoalliin (S-1-propenyl-L-cysteine sulfoxide) [6].

Organic sulfides are one of the main bioactive components contained in plants, and the consumption of sulfur-containing vegetables has a positive effect on human health [7]. *Allium* is known for its rich organic sulfides [8]. The main organic sulfides in *Allium* are allyl cysteines, S-alk(en)yl-L-cysteine sulfoxide (ACSO) and thiosulfinates [9]. In addition to organic sulfides, polyphenols are other important bioactive compounds in *Allium*, especially phenolic acids, flavonoids and their derivatives [10]. Leeks are one of the richest sources of flavonoids in the human diet. The main flavonoids of onion are quercetins and their conjugates [11]. Different polyphenols have different sugar units and acylated sugars at different positions on their main chains. At present, polyphenols are widely used in health food and medicine because of their beneficial impact on various biological functions and their lack of side effects on human body [12,13]. In recent years, with the study and utilization of the active substances of *Allium* plants, it has been found that most of them have antioxidant, anti-tumor, antibacterial and lipid-lowering effects, as well as contributing to cardiovascular disease prevention and other biological functions [2,14,15,16]. However, the current research on *Allium* mainly focuses on the biological functions of garlic and onion [17,18]. Given that leeks are a common vegetable on Asian tables, with rich nutritional value and unique flavor qualities, their active components and biological functions have been comprehensively summarized and updated in this review.

**Figure 1:**
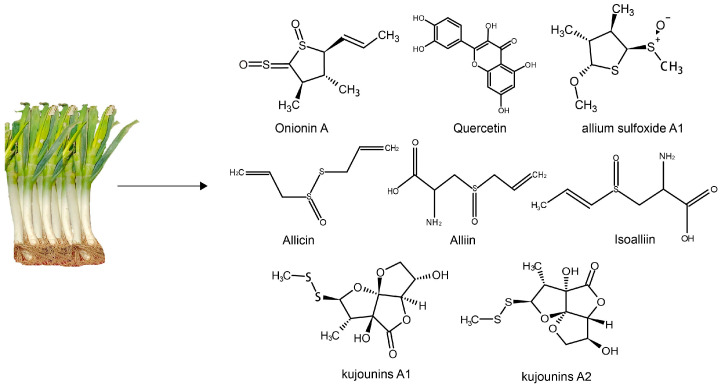
The chemical structures of the main sulfides and phenolic compounds in leeks.

Leek (*Allium fistulosum* L.) is a common food component in *Allium Liliaceae*. It is mainly used as a traditional food seasoning and is widely consumed around the world. Its stem is short and globose or oblate and is surrounded by the base of the leaf sheath. The roots are stringy, and the lateral roots are few and short. The aboveground part is yellowish-green, and the underground part is white. The overall appearance is cylindrical, with long yellow-green leaves and a smooth and waxy surface. In 2015, Zhang et al. [19] found a variety of bioactive compounds in leeks, including sulfides, polyphenols and dietary fibers (see Figure 1). 

**Figure 2:**
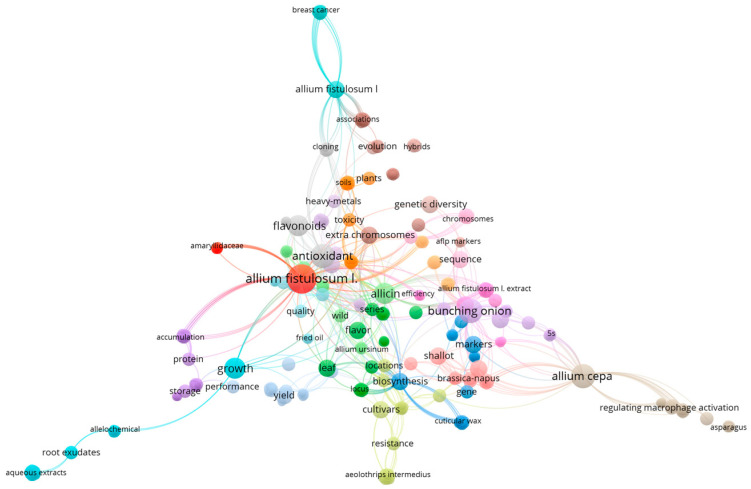
Cluster analysis of the literature research on *Allium fistulosum* L.

In order to gain a deeper understanding of the current state of research on leeks, the Web of Science database searched for the literature on leeks. Entering *Allium fistulosum* L. into the Web of Science core collection yielded a total of 220 publications. All the literature was imported into a VOSviewer_1.6.19 literature processing software for analysis. After the literature removal and data cleaning, we compiled the results of the cluster analysis of the leek literature studies as shown in Figure 2. The sizes of the different colored circle areas in the figure represent the amount of relevant literature and the intensity of research in different directions. As shown in the figure above, studies on the bioactive functions of leeks are relatively few, focusing mainly on the antioxidant level of leeks, and to a lesser extent on their effects of anti-inflammation, anti-bacteria [20], anti-cancer [21], lipid-lowering [22], anti-hypertension and prevention of cardiovascular diseases [23,24,25]. The trend of research also changed from the study of crude extracts of different parts of leeks to the study of the biological effects and mechanisms of different active monomers, as well as the biosynthesis of these bioactive components. In this paper, the research progress of the active components and their biological functions as found in leeks were systemically reviewed. 

## 2. Characteristics and Composition of Leeks

Leeks are a type of green onion vegetable and one of the most common species on the table. They have the characteristics of high yield, resistance to decomposition during storage and transportation and high economic value. According to the botanical classification standard, leeks mainly include three species groups, including Chinese, Japanese and Russian varieties. These different varieties of leeks vary in stem length, leaf size, color, sweetness and spicy flavor [26]. China is a major producer of leeks. The planting of leeks is mainly distributed in the Huaihe River basin, the north of the Qinling Mountains and the middle and lower reaches of the Yellow River. The water content of leeks is 92~95%, the protein content is 1.42~1.49% and the carbohydrate content is 2.02~5.64% [27]. There are also 35 kinds of trace elements (barium, beryllium, bismuth, cadmium, cobalt, chromium, copper, etc.), 17 amino acids (aspartic acid, threonine, serine, glutamic acid, glycine, alanine, etc.) and fatty acids in leeks [28]. These nutrients give leeks their unique pharmacological properties.

Leeks have a distinctive, strong and pungent smell, which is produced by the volatile oils. The main component of volatile oil is sulfide, accounting for 90.45% of the total volatile substances, and the other main components are fatty acids and nitrogen compounds [29]. Organic sulfides in leeks have sulfur atoms attached to cyanate groups or carbon atoms with annular or non-annular structures. Leeks contain a non-volatile precursor compound called alliin. Alliin exists in an unstable form. When leeks are sliced or crushed, the cellular structure within them is damaged, and allinase separates from the substrate, activating the precursor alliin to form allicin, which is broken down to produce the strong-smelling sulfide [30]. It has been pointed out that allicin is the parent sulfur compound. The flavor and medicinal value of *Allium* are mainly due to the hydrolytic reaction between allicin and allinase during the occurrence of tissue damage, and the cleavage of the C-S bond from allicin. The resultant sulfonic acid is unstable and spontaneous reactions occur which produce a variety of sulfur-containing compounds [31]. Allinase is present in the vacuoles of all *Allium* plants. Only after the tissue breaks down does the catalytic reaction occur. And the first precursor compounds formed are sulfonic acid and thiosulfonic salts, which are the intermediates that form most of the sulfur volatiles [32]. After the extraction and separation of leek leaves (1.1 kg), the obtained sulfides were onion A1 (34.2 mg), onion A2 (22.1 mg) and onion A3 (16.4 mg) [33]. In addition, sulfur compounds in leeks include matrine A1, A2 and garlic sulfide A1 [34]. Taken together, these sulfides of active compounds in leeks are closely related to different biological activities.

## 3. Biological Functions of Leeks

The bioactive ingredients extracted from leeks could effectively provide anti-inflammatory, antioxidant, anti-cancer and other protective effects. They also have a preventive and antihypertensive effect on the cardiovascular system. In addition, they can also produce an anti-microorganism and anti-viral effect. The biological functions of extracts obtained from leeks by different extraction methods are listed in Table 1.

### 3.1. Anti-Inflammatory Activities

**Figure 3:**
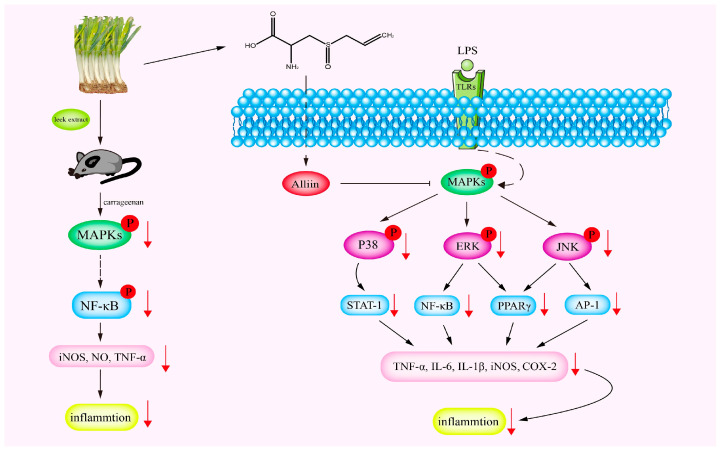
Potential mechanisms of the anti-inflammatory effect of leeks. Under stressful conditions, leek extract offsets the inflammatory response by inhibiting the MAPK signaling pathway. MAPK: mitogen-activated protein kinase; NF-κB: nuclear factor kappa-B; STAT-1: signal transducer and activator of transcription 1; PPARγ: peroxisome proliferator-activated receptors γ; AP-1: activator protein 1; TNF-α: tumor necrosis factor alpha; IL-6: interleukin 6; IL-1β: interleukin-1β; iNOS: inducible nitric oxide synthase; COX-2: cyclooxygenase 2; NO: nitric oxide.

Inflammation is a collective defensive response to stimuli. It is a normal biological response produced by the human body to resist the damage of infected tissues and harmful stimuli from the outside. It usually presents with symptoms such as redness, swelling, heat pain and dysfunction. These symptoms are induced by cytokines and other inflammatory mediators [51]. Cytokines can be divided into anti-inflammatory cytokines and pro-inflammatory cytokines, according to their effect. Anti-inflammatory cytokines, including IL-4, IL-6, IL-10 and TGF-β, could inhibit inflammation. In contrast, pro-inflammatory cytokines, including IL-1β, IL-6, TNF-α and interferon, could promote inflammation and stimulated immunoactive cells [52]. Pro-inflammatory cytokines could enhance the immune capacity of the body. Inflammation is the automatic defense of the body, and it is an organism’s defensive response to invading pathogens or endogenous signaling stimuli to clear dead cells and carry out tissue repair [53]. Inflammation can be harmful, and it can attack the body’s own tissues. The anti-inflammatory activity of leek extracts are well documented. Alam et al. [35] found that the extracts from leeks showed significant inhibition of NO (nitric oxide) production in lipopolysaccharide (LPS)-activated macrophages in a dose-dependent manner. NO, an important mediator for inflammation, is mainly produced by macrophages in the initiation stage of dynamic atherosclerosis [54]. During inflammation, innate immune cells produce relatively high levels of NO [55]. Leek extracts showed a strong inhibitory effect on the NO production of RAW264.7 cells induced by LPS, with half inhibitory rates of 2.01 ± 1.40 μm and 2.49 ± 1.54 μm, respectively [56]. Tsai et al. [36] analyzed the anti-inflammatory activity of leeks based on their ability to inhibit NO production in LPS-activated RAW264.7. When the cells were treated with leek extracts and LPS, a significant concentration-dependent inhibition of NO was detected. An increase in the activity of inflammatory enzymes leads to an increase in NO, nitric oxide synthetase (NOS) and cyclooxygenase-2 (COX-2). These could contribute to the occurrence of inflammation and the impairment of cell and tissue function caused by inflammation [57,58]. Similarly, the anti-inflammatory effects of the water extracts of leeks were verified by in vivo tests on mice. In the range of 0.25 g/kg~1 g/kg, the extracts improved carrageenan-induced edema and lipid oxidation. These are often thought to be a result of inflammation in the body. In addition, the serum nitrite levels and serum TNF-α levels of the carrageenan treatment group were decreased by 17–53% and 24–51%, respectively, after treatment with leek extracts. The experiment demonstrated that the water extracts of leeks can inhibit inflammation in mice by reducing the release of cytokines [37]. Many studies have confirmed that, in the process of inflammation, the expression levels of NO, TNF-α, COX-2 and iNOS are increased. Another study explored the anti-inflammatory effect of leek extracts on LPS-stimulated BV2 microglia [38]. Four different extraction methods used to extract leeks were studied, and it was found that all four extracts could effectively inhibit the mRNA and protein expression levels of iNOS and COX-2 at the cellular level. In addition, the production of pro-inflammatory factors including TNF-α, IL-6 and IL-1β also decreased significantly at mRNA level. Consequently, the anti-inflammatory effect of leeks could be verified by observing the expression levels of these cytokines. Further study on the anti-inflammatory activity of leeks showed that the significant anti-inflammatory activity of leek extracts may be related to allicin contained in leeks. Alliin is a compound isolated from leeks which is produced by enzymatic reaction after tissue destruction. Alliin could reduce intestinal inflammation by inhibiting the activation of the MAPK-NF-κB pathway [59,60,61,62]. These studies show that Welsh onion and its active components have anti-inflammatory activity. It should be noted that the anti-inflammatory mechanism of Welsh onion may be related to the inhibition of MAPK and NF-κB activation, the reduction in proinflammatory cytokine and the enhancement of anti-inflammatory cytokines. The anti-inflammatory mechanisms of leeks and their bioactive components are shown in Figure 3.

It is known that both iNOS and COX-2 expressions are regulated by nuclear κB transcription factor (NF-κB) [57,58,63]. NF-κB is a transcription factor involved in apoptosis, tumorigenesis, and inflammation. It is composed of homologous or heterodimers of different subunits. Activation of NF-κB is associated with various chronic inflammations. The classic NF-κB pathway is activated by pro-inflammatory signals such as IL-1R and the TNF receptor (TNFR) family. Therefore, many drugs targeting anti-inflammation act by inhibiting the NF-κB pathway [63]. IκB is an inhibitor of NF-κB that inhibits the activation of NF-κB in unstimulated cells. In response to stimulation, NF-κB is activated by phosphorylation of the IκB protein [64]. Wang et al. [39] studied the effect of the green leaf extract of leeks on NO production in macrophages. When the leek extract was added to RAW264.7 cells at a concentration of 1.0 mg/mL, the production of NO was completely inhibited and NO-induced DNA damage and cytotoxicity was avoided; 0.5 mg/mL extract could down-regulate the expressions of iNOS and COX-2 in the RAW 264.7 cells. The activation of NF-κB is the main response to LPS-induced iNOS and COX-2 expression in inflammatory cells. Western blotting analysis showed that the extract may inhibit the activation of NF-κB by up-regulating the expression of IκB-α protein. Therefore, green leaf leek extract may exert its anti-inflammatory effects by preventing the activation of NF-κB and inhibiting the expressions of iNOS, COX-2 and the production of NO. In general, leeks have been found to be effective in relieving inflammation. The anti-inflammatory mechanism of leeks may be mediated by inactivating the NF-κB pathway and inhibiting the expression of iNOS and COX-2. However, because of the complexity of inflammation-related signaling pathways, whether leeks could also play an anti-inflammatory role through other targets and signaling pathways is still unclear. 

### 3.2. Anti-Cancer Activities 

Cancer is one of the leading causes of death in the world. It is also a key obstacle to increasing life expectancy [65,66]. Some studies have shown that dietary changes can have a significant effect on improving cancer treatment, and that healthy eating habits can reduce the risks of cancer [67,68,69]. Natural products derived from plants have attracted considerable attention for their potential benefits as chemotherapy agents and prophylactic agents against cancer [70]. At the same time, the discovery of some new targets for these natural products in order to prevent or treat tumors plays an important role in cancer therapy [71]. For example, the regulation of mTOR, AMPK, MAPK and other signaling pathways can achieve the effect of cancer prevention and even treatment, and these signaling pathways can be targeted to find suitable active substances in natural products [71,72,73]. In recent years, *Allium* plants have attracted much attention for their effects on various diseases, especially tumors. In addition, they are able to alleviate the side effects of current anti-cancer drugs, which are closely related to their bioactive compounds, such as sulfur compounds, flavonoids and saponins [1,35,74]. Studies found that the consumption of *Allium* vegetables is significantly associated with a reduced risk of various cancers, such as colorectal cancer [21,75], liver cancer [76] and breast cancer [42]. It has been confirmed that human breast cancer cells (MDA-MB-453) were treated with 100 μg/mL leek extract for 24 h, which significantly inhibited cell proliferation [77].

Different leek extraction methods had different inhibitory effects on tumors. Arulselvan et al. [21] evaluated the effect of leeks on colorectal cancer. In the CT-26 tumor-bearing mice, both hot and cold water extracts and ethanol extracts of leeks were found to inhibit tumor growth and induce tumor cell apoptosis. After 17 days, hot water extracts of leeks showed the highest inhibitory rate on tumor growth. From the perspective of a molecular mechanism, the hot water extracts of leeks can inhibit the protein expression levels of cyclin D1, c-Myc, MMP-9, ICAM, VEGF and HIF-1α. Moreover, the protein expression levels of some inflammatory cytokines, such as iNOS, COX-2, IL-6 and TNF-α, were down-regulated. These results suggest that hot water extracts of leeks produce anti-cancer effects by inhibiting activities related to cell proliferation, angiogenesis, inflammation and inducing apoptosis. Different cancer cell lines have different sensitivities to allicin. The IC50 value of DLD-1 cells, which were most sensitive to the leek extracts, was 2.124%, and the IC50 values of MDAM231, MCF7, and SK-MES-1 were 2.464%, 3.353% and 5.819%, respectively. These results proved that the extracts of leeks produced a dose-dependent inhibitory effect on tumors. From the point of view of a molecular mechanism, the extracts of leeks can induce cell apoptosis mainly by increasing the ratio of Bax/Bcl-2 and enhancing the activity of Caspase-3 [40]. Leeks extracted by different extraction methods had different inhibitory effects on tumors and could inhibit tumor growth and induce tumor cell apoptosis.

**Figure 4:**
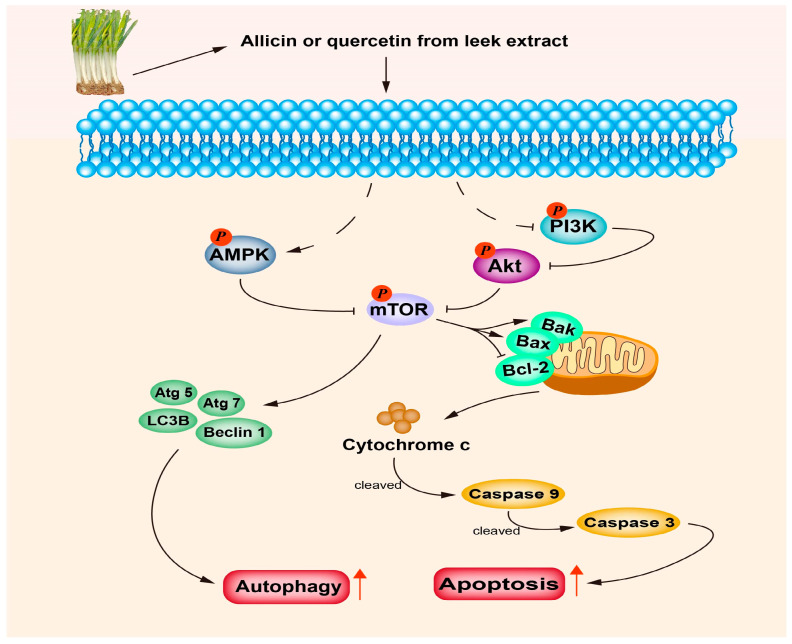
The anti-cancer mechanisms of leeks and their active compounds. Allicin or quercetin isolated from *Allium* extract exerts its anti-cancer effects mainly through the following pathways: activating apoptosis and autophagy mediated by AMPK/mTOR and PI3K/Akt/mTOR signaling pathways, changing the expressions of Bax/Bcl-2, releasing cytochrome c from mitochondria and increasing cleaved Caspase 9 and Caspase 3, thus promoting the apoptosis of cancer cells. Inhibition of mTOR can significantly promote the expressions of Atg 5, Atg 7, Beclin1 and LC3B, which can promote the autophagy of cancer cells.

The abundance of bioactive substances in leeks is also one of the reasons why leeks produce anti-cancer activity. Allicin induced the activation of Caspase-3, Caspase-8, Caspase-9 and the loss of mitochondrial membrane potential in human breast cancer cells MCF-7 and HC-70. Furthermore, some pro-apoptotic genes (p21, NOXA, Bak) were up-regulated and anti-apoptotic genes (Bcl-xl) were down-regulated after being treated with allicin, thus inducing apoptosis of MCF-7 and HC-70 [78]. In recent years, a series of studies have been conducted on the anti-proliferative effects of phenolic compounds on malignant tumors in vitro and in vivo. At present, quercetin and isoquercitrin are mainly studied for the phenolic compounds contained in leeks. In terms of the molecular mechanism, phenolic compounds, especially quercetin, induce apoptosis mainly by reducing the expression of the anti-apoptotic proteins Bcl-2 and Bcl-xl and increasing the expression of the pro-apoptotic protein Bax, so as to achieve the anti-cancer effect [41]. Quercetin is the main representative of the flavonoid subclass of flavanols, which mainly exists in the form of glycosides in food. After being ingested by the human body, glycosides are hydrolyzed, thus releasing glycoside for absorption and metabolism by the human body [79]. Some studies have used high-performance liquid chromatography (HPLC) to determine the content of quercetin in leeks, and the human hepatocellular carcinoma cell line was treated by quercetin. It was found that quercetin had a significant inhibitory effect on the human hepatocellular carcinoma cells HepG2, but not on the human colorectal cancer cells HT-29 or human prostate cancer cells PC-3 [80]. Quercetin could cause G1 phase arrest in breast cancer cells and inhibited cell proliferation. In addition, quercetin induces tumor cell apoptosis, autophagy and reduces tumor cell viability mainly by reducing the stability of β-Catenin and HIF-1α, activating the expression of caspase 3 and inhibiting the phosphorylation of Akt, mTOR and ERK [42]. Generally, leek extract or its main active components have strong cytotoxicity against breast cancer, liver cancer and colorectal cancer. The mechanism mainly involves the inhibition of cancer cell proliferation and the induction of cell apoptosis (see Figure 4). Flavonoids represented by quercetin have good anti-cancer effects, while other active ingredients in leek extract also have anti-cancer effects; in addition, leek extract is rich in polyphenols, and whether the anti-cancer effect of leeks is also related to polyphenols needs to be studied further.

### 3.3. Antioxidant Activities

Reactive oxygen species (ROS) are first introduced into skeletal muscle as free radicals, mainly including superoxide anion (O^2−^), hydroxyl radical (-OH), peroxyl radical (HO2) and NO [2]. Subsequent studies have found that the ROS functions in cells range from aiding immunity to acting as signaling molecules [81]. ROS regulate various signaling pathways mediated by transcription factors NF-κB and STAT3, kinases, cytokines and enzymes, which are involved in inflammation, tumor survival, proliferation, invasion, angiogenesis and metastasis [82]. ROS are the main causes of oxidative stress, and the enhanced oxidative stress state can cause a series of chronic diseases, such as cancer, diabetes, coronary heart disease and vascular diseases [83]. Therefore, the most direct and effective way to reduce oxidative stress is to remove the accumulation of free radicals in the body and inhibit the production of free radicals [84]. Antioxidants are substances that inhibit oxidation and avoid or eliminate the risk of oxidative stress-induced metabolic disorders and related diseases by resisting the deterioration of ROS [85]. 

The antioxidant potential of leeks is mainly derived from its flavonoids and organosulfur compounds. Flavonoids have the ability to scavenge O^2−^ and NO radicals, and their antioxidant activity is mainly determined by the number and configuration of B-ring hydroxyl groups [43]. Flavonoids in leeks, including quercetin and quercetin glycosides, have been reported to be closely related to their anti-cancer and antioxidant properties [80,86]. EI-Hadidy et al. [87] found that three main flavonoids were isolated from leek leaves, namely myricetin, quercetin and rutin, and their contents were 38.75%, 11.43% and 11.27%, respectively. The antioxidant activity of leek leaves was determined by the DPPH radical scavenging assay. It was found that after three months of storage, the antioxidant activity of the leek leaves decreased with the decrease in antioxidant percentage. The antioxidant capacitis of leeks are highly correlated with its total phenolic content. Studies have shown that phenolic compounds may be the main supporters of antioxidant activity in leeks. In addition, the extracts of leeks treated with higher nitrogen levels showed higher total phenol content and antioxidant levels [88]. Medina-Jaramillo et al. [89] found that ferulic acid and p-coumaric acid were the most abundant when using UHPLC-ESI+-Orbitrap-MS to analyze the phenolic acids in the extracts of leek leaves, and the ability of the extracts to scavenge DPPH radicals increases with the increase in ethanol concentration and extraction time. Therefore, the antioxidant capacity of the extracts of leek leaves is closely related to external factors. In another study, the antioxidant activities of wine extracts from different parts of leeks were evaluated. The results showed that the allicin content in the wine extracts of leeks ranged from 28.3 μL/mL to 95.9 μL/mL. In addition, the DPPH radical scavenging ability of the wine extracts from different parts of leeks ranged from 52.1% to 90.2%, IC50 values was ranged from 14.6 μg/mL to 26.0 μg/mL, and TEAC values ranged from 6.2 mmol/g to 15.5 mmol/g. Under different extract methods, the wine extracts of leeks had higher allicin content and DPPH free radical scavenging ability, IC50 and total antioxidant capacity were satisfactory [44]. It is reported that vitamins A and C, carotenoids and chlorophyll contained in leeks also contribute to its antioxidant capacity [90]. Antioxidant effects of leeks were accompanied by decreased NADH/NADPH oxidase activity in vascular tissues and increased levels of NO metabolites in urine and plasma [23]. Studies have confirmed that leek extract has antioxidant activity, which could resist oxidative damage under various conditions and has protective effects on vascular endothelial cells and ischemia reperfusion after myocardial infarction. At the same time, it was found that leek extract could inhibit xanthine oxidase activity and clear NO, O^2−^, -OH and metal ion chelating agents [43]. Wang et al. [39] studied the effect of a water extract of leeks’ green leaves on the antioxidant activity of RAW264.7 cells. The results showed that the protective effect of the extract on Cu^2+^ induced low-density lipoprotein (LDL); oxidation increased with the increase in the extract concentration of 0.1~1.0 mg/mL. When the concentration reached 1.0 mg/mL, it had absolute inhibition on Cu^2+^-induced LDL oxidation. With further study, it was found that the extract inhibited the production of NO, iNOS and COX-2 proteins by blocking NF-κB in RAW264.7 stimulated by LPS. The total of the antioxidant effects of green leaf extract from leeks were determined by TEAC method. Finally, the experiment found that the total antioxidant activity increased with the increase in solution concentration in the range of 0.01 mg/mL–1.0 mg/mL. In conclusion, leeks and their active constituents (such as phenols and flavonoids) have certain antioxidant effects. The antioxidant activities of leeks and their active constituents were verified by scavenging DPPH, OH, O^2−^ free radicals and metal ion chelating agents. Different extraction methods also affect the antioxidant activity of leeks. In general, the antioxidant activity of leeks extracted with ethanol is stronger than that of water. In addition, in vitro experiments showed that the mechanism of the antioxidant action of leeks may be related to the increase in antioxidant active enzymes.

### 3.4. Anti-Obesity Activities

Obesity is caused by an imbalance between the amounts of calories consumed and the amount of energy expended, which promotes the expansion of fat tissue that is needed to buffer excess nutrients. Obesity is an important cause of many chronic diseases, such as diabetes, hypertension and cardiovascular disease [91,92]. Studies have shown that leek extract could reduce obesity caused by a high-fat diet, and leeks were effective in the management and prevention of obesity [46]. The evidence and mechanism of the anti-obesity effect of leek extract can be verified by in vivo experiments.

In an in vivo experiment, mice on a high-fat diet were fed 70% ethanol extract of leeks. The results showed that both the weight of white adipose tissue and the size of fat cells were significantly inhibited, and the mice lost about 10% of their body weight. The levels of serum lipid parameters were observed in the follow-up experiment. It was found that the extracts significantly inhibited the increase in triglyceride, total cholesterol and low-density lipoprotein cholesterol concentrations and increased the serum adiponectin concentration. From the point of view of a molecular mechanism, mRNA expression levels of transcription factors such as SREBP1c, PPARγ and FAS in the subcutaneous tissues of mice treated with an ethanol extract of leeks were decreased compared with those in the high-fat diet group [22]. Yamamoto et al. [45] found that adding leeks to the diet could reduce the weight of rats fed with a high-fat, high-sugar diet. The possible mechanism was to effectively reduce the lipid level in the liver and plasma of rats by affecting hepatic fatty acid metabolism. In another study, leek extracts also reduced liver fat accumulation, adipocyte size and body weight in mice fed with a high-fat diet. The expression levels of IGF-1 and PPAR-γ were down-regulated, and the mRNA expression levels of AMPK-α in the liver and UCP-2 and lipocalin in adipose tissue were increased after treatment with leek extract [46]. In addition, an in vitro experiment verified the effect of leeks on reducing blood lipids. HepG2 cells were cultured in a medium with degreasing serum under lipid depletion condition. After adding the extract of leeks (70% ethanol), it was found that the extract of leeks decreased the expressions of lipid metabolism-related genes, such as Ldlr, PCSK9 and HNF1α, by decreasing the expression of SREBP2 [47]. Leek extract can reduce the effects of a high-fat diet on body weight and adipose tissue in mice and have an anti-obesity effect. Although some molecular mechanisms of its lipid-lowering effect have been explored, further studies are needed. Taken together, leeks have a certain positive effect on obesity by inhibiting adipogenesis, reducing fat accumulation and regulating fat metabolism.

### 3.5. Antibacterial Activities

The advent of antibiotics has given new hope to some patients with infectious diseases. However, a new crisis has emerged due to the overuse of antibiotics and antibiotic-resistant pathogens. The World Health Organization (WHO) has identified antibacterial resistance as one of the top 10 global public health threats to humanity [93]. As a result, there is growing interest in finding natural compounds that can replace antimicrobials.

**Figure 5:**
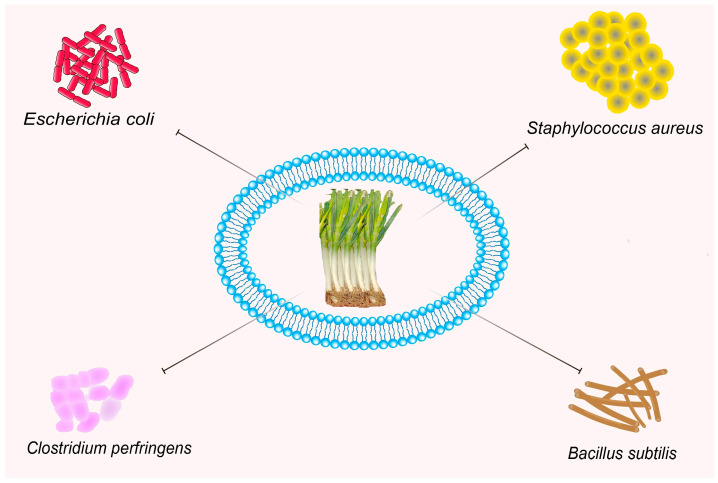
The antibacterial effects of leeks.

Some evidence has been published regarding the antibacterial properties of leeks and some of their components. In 2015, Zhai et al. [48] evaluated the antibacterial activity of leeks. The biological characteristics of 16 secondary metabolites isolated from the stems of leeks were characterized using Gram-positive bacteria and Gram-negative bacteria. Compound 1 was shown to have antibacterial activity against both *Clostridium perfringens* and *Bacillus subtilis* by a micro dilution method in a biological screen with minimum inhibitory concentrations of 12.5 μg/mL and 6.25 μg/mL, respectively. In addition, both compound 2 and compound 11 showed significant antibacterial activity against *Escherichia coli*. In another experiment, the phenolic compounds isolated from leeks were determined to produce an inhibitory effect on the growth of *Escherichia coli* and *Staphylococcus aureus*. *Staphylococcus aureus* was more sensitive to the phenolic compounds in the tested leeks than *Escherichia coli* [49]. Silver nanoparticles synthesized using leeks exhibited excellent inhibition of both bacterial and fungal strains [94]. It has also been suggested that the inhibition of microorganisms by leeks is mainly due to the action of components within the fat-soluble substances in leeks [50]. These studies show that leek extract has certain antibacterial activity (see Figure 5). However, it is worth noting that the antibacterial activity of leek extract is still in the initial stage; however, which active components in leeks play a major role and the mechanism they use is not clear. Taken together, there are few studies on the antibacterial activity of leeks, so the evidence on its antibacterial performance is limited. In-depth studies on the molecular mechanisms underlying the antibacterial activity of leeks should also be conducted in future studies.

### 3.6. Other Functions

In addition to the above biological activities, studies have proven that leeks have a protective effect on drug-induced hepatotoxicity in rats. Cha et al. [95] evaluated the effect of leeks on CCl_4_-induced liver injury in rats. The results showed that leeks could significantly reduce serum aminotransferase (SGOT and SGPT) and alkaline phosphatase (ALP) levels. In addition, the leeks produced in winter showed more significant liver protective activity, at 4% and 10% (*w*/*w*) doses, and had a certain preventive effect on CCl4-induced inflammation and vacuole. Leeks have also shown therapeutic effects in preventing cardiovascular disease. For example, leek extract can change the function of human platelets. The effects of crude extract and cooked extract of leeks on platelets were studied. The results showed that the crude extract of leeks inhibited ADP-induced platelet aggregation, platelet adhesion on the fibrinogen map surface and thromboxane production, while the cooked extract did the opposite. One of the beneficial effects on the cardiovascular system is inhibition of platelet activity, such as platelet aggregation and release [25]. Yamamoto et al. [23] investigated the effect of leeks on hypertension in rats fed with a high-sugar and high-fat diet. After the rats were fed for four weeks, the effects of garlic bulb extracts on rats were observed. The rats were observed after four weeks of continuous feeding with the bulb portion extract of leeks. The results showed that systolic blood pressure and malondialdehyde concentration were increased in the rat with a high-sugar and high-fat diet. Supplementation of leek extract could inhibit angiotensin II production and the activity of NADH/NADPH oxidase and reduce the synthesis of superoxide; meanwhile, supplementation of leek extract also promoted NO production, thereby reducing the blood pressure of rats in the hypertension animal model. In addition, the extract of leeks was used to treat letrozole-induced rats. It showed improvement in the performance of aromatase, relieved the obstruction of testosterone conversion to estrogen, increased estrogen to balance hormone levels in the body and improved the ovarian function of rats [96]. In 2019, Yang et al. [97] reported that ovariectomized rats fed with rice porridge containing a water extract of leek roots relieved pain behavior and bone metabolism associated with osteoarthritis. Lee et al. [98] isolated a fructan from the hot water extract of the green leaf parts of leeks, and oral administration of this fructan inhibited the replication of influenza A virus in vivo. Although the biological functions of leeks are complex, the good health effects of green onion cannot be underestimated. At present, people have discovered the complex, positive health function of leeks. In the future, it is still necessary to deepen the research on the biological activity function of leeks and study the relationship between different activity functions to maximize the biological function of leeks. 

## 4. Conclusions

In summary, leeks contain a variety of bioactive substances, such as allicin, quercetin, and other organic sulfides. Leeks have a variety of biological active effects, such as anti-cancer, anti-inflammation, anti-obesity, anti-oxidation and anti-bacteria, but the action mechanisms of these biological effects are still unclear. For example, can leeks affect gut microbiota and metabolic products? How are these metabolites involved in different biological functions? Existing studies have identified some important target genes and associated signaling pathways for leeks’ active components, but are there other target genes involved in these biological effects? The application of new technologies, such as transcriptomics, proteomics and metabolomics, will help to understand the molecular mechanism of its function. In addition, there may be synergies between different active compounds of leeks and the cross-talk of signal pathways might exist. Meanwhile, the effects of heating and other processing methods on leeks need to be studied further in order to explore their effects on biological functions. Clinical trials need to be conducted to confirm the health benefits of leeks. Further study of their active components and mechanisms of action will contribute to this as well.

## Figures and Tables

**Table 1:** Biological activities of leek extract.

Bioactivities	Extraction Method (Extract)	Model	Molecular Mechanisms	References
Anti-inflammatory activities	The aqueous extract of *A. fistulosum*	LPS-activated macrophages	NO↓	[35]
	The aqueous extract of *A. fistulosum*	LPS-activated RAW 264.7	NO↓, iNOS↓	[36]
	The water extract of *A. fistulosum*	Carrageenan-induced edema in mice	NO↓, iNOS↓, TNF-α↓	[37]
	Water extract from the whole *A. fistulosum* Ethanol extract from the whole *A. fistulosum* Water extract from the root of *A. fistulosum* Ethanol extract from the root of *A. fistulosum*	LPS-stimulated BV2	iNOS↓, COX-2↓, TNF-α↓, IL-6↓, IL-1β↓	[38]
	Aqueous extracts of Welsh onion green leaves	LPS-activated RAW 264.7	NF-κB↓, NO↓, iNOS↓, COX-2↓	[39]
Anti-cancer activities	Hot water extract of scallion Cold water extract of scallion Ethanol extract of scallion	A mouse model of colon carcinoma (CT-26 cells)	cyclin D1↓, c-Myc↓, MMP-9↓, ICAM↓, VEGF↓, HIF-1α↓, iNOS↓, COX-2↓, IL-6↓, TNF-α↓	[21]
Anti-cancer activities	Aqueous extracts of Welsh onion	DLD-1, MDA-MB-231, MCF-7, SK-MES-1	Bax↑, Bcl-2↓, Caspase 3↑	[40]
	Alliin, Allicin, Quercetin	MCF-7 and HCC-70	Caspase 3↑, Caspase 8↑, Caspase 9↑, P21↑, NOXA↑, Bax↑, ΔΨm↓, Bcl-xl↓	[41]
	Quercetin	Breast cancer cell lines (MCF-7, MDA-MB-231, HBL100 and BT549)	β-catenin↓, HIF-1α↓, Caspase 3↑, p-Akt↓, p-mTor↓, p-ERK↓	[42]
Antioxidant activities	Water-soluble extract of Welsh onion Lipid-soluble extract of Welsh onion	Rats fed a high-fat, high-sucrose diet	angiotensin II↓, NADH/NADPH↓, TBARS↓,	[23]
	Aqueous extract of Welsh onion green leaves	—	NO↓, O^2−^↓, ·OH↓	[43]
	Aqueous extract of Welsh onion green leaves	Macrophage cell line (RAW 264.7)	LDL↓, NF-κB↓, NO↓, iNOS↓, COX-2↓	[39]
	Rice wine extracts of Taiwanese *Allium fistulosum*	DPPH ethanolic solution; ABTS solution diluted with water	—	[44]
Anti-obesity activities	70% ethanol extract from *Allium fistulosum* L.	High-fat, diet-induced obese mice	SREBP1c↓, PPARγ↓, FAS↓	[22]
	Welsh onions were crushed and heated to denature enzyme activity, freeze dried, and ground	High-fat, high-sugar fed rats	—	[45]
Anti-obesity activities	The aqueous and ethanolic extracts from the *Allium fistulosum*	High-fat, diet-induced obese mice	IGF-1↓, PPAR-γ↓, AMPK-α↑, UCP-2↑	[46]
	70% ethanol extract from Welsh onion	HepG2 cells	SREBP2↓, LDLR↓, PCSK9↓, HNF1α↓	[47]
Antibacterial activities	Extraction separation	Gram-positive and Gram-negative (Candida cyclic, Bacillus subtilis, *Escherichia coli* and *Staphylococcus aureus*)	—	[48,49,50]

## Data Availability

Data is contained within the article.

## Abbreviations

Akt	Protein kinase B
AMPK	Adenosine 5′-monophosphate (AMP)-activated protein kinase
Bax	Bax
Bcl-2	B-cell lymphoma-2
Bcl-xl	B-cell lymphoma xl
Caspase	Cysteinyl aspartate-specific proteinase
CCl4	Carbon tetrachloride
COX-2	Cyclooxygenase-2
ERK	Extracellular signal-regulated kinase
FAS	Recombinant factor-related apoptosis
HIF-1α	Hypoxia inducible factor-1α
ICAM	Intercellular cell adhesion molecule
IL-10	Interleukin-10
IL-4	Interleukin-4
IL-6	Interleukin-6
iNOS	Induced nitric oxide synthase
LDL	Low-density lipoprotein
LPS	Lipopolysaccharide
MMP-9	Matrix metalloprotein 9
mTOR	Mammalian target of rapamycin
NADH	Nicotinamide adenine dinucleotide
NADPH	Nicotinamide adenine dinucleotide phosphate
NF-κB	Nuclear kappa B
NO	Nitric oxide
PCSK9	Proprotein convertase subtilisin/kexin type 9
PPARγ	Peroxisome proliferator-activated receptors γ
ROS	Reactive oxygen species
SREBP1c	Sterol regulatory element binding protein 1c
SREBP2	Sterol regulatory element binding protein 2
STAT3	Signal transducer and activator of transcription-3
TGF-β	Transforming growth factor β
TNF-α	Tumor necrosis factor α
UCP-2	Uncoupling protein 2
VEGF	Vascular endothelial growth factor
